# The effect of dietary tryptophan levels on oxidative stress of liver induced by diquat in weaned piglets

**DOI:** 10.1186/2049-1891-5-49

**Published:** 2014-11-04

**Authors:** Xiangbing Mao, Mei Lv, Bing Yu, Jun He, Ping Zheng, Jie Yu, Quyuan Wang, Daiwen Chen

**Affiliations:** Animal Nutrition Institute, Sichuan Agricultural University, Xinkang Road 46#, Ya’an, Sichuan Province 625014 People’s Republic of China; Key Laboratory of Animal Disease-Resistance Nutrition, Ministry of Education, Ya’an, 625014 People’s Republic of China

**Keywords:** Antioxidant capacity, Diquat, Oxidative stress of livers, Tryptophan, Weaned piglets

## Abstract

Oxidative stress can induce abnormal tryptophan metabolism. The present study was mainly conducted to determine the effect of dietary tryptophan levels on oxidative stress in the liver of weaned pigs challenged by diquat. A total of 36 PIC piglets weaned at 21 days of age were randomly allotted to 1 of 3 diets containing dietary tryptophan levels of 0.18, 0.30, and 0.45% for 14 d. On day 8, the piglets were injected intraperitoneally with sterile 0.9% NaCl solution or diquat (10 mg/kg body weight). During the first 7 d of trial, increasing dietary tryptophan levels enhanced average daily gain (*P* = 0.09) and average daily feed intake (*P* = 0.08), and decreased the feed efficiency (*P* < 0.05) of piglets. The growth performance was decreased by diquat injection (*P* < 0.05). Diquat injection also decreased the activities of the superoxide dismutase (SOD) and glutathione peroxidase (GPx) in the plasma and liver (*P* < 0.05), increased plasma malondialdehyde (MDA) (*P* < 0.05) and urea nitrogen (*P* < 0.05) concentrations, and enhanced MDA concentration (*P* = 0.09) and tryptophan 2,3-dioxygenase (TDO) activity (*P* = 0.07) in liver of piglets. Increasing dietary tryptophan levels could attenuate the effects of diquat injection on the MDA (*P* = 0.06) concentration and the activities of SOD (*P* = 0.09) and GPx (*P* = 0.05) of the liver, and plasma urea nitrogen (*P* = 0.06) concentration in the piglet. There was a synergistic role for increasing TDO activity in the liver between dietary tryptophan levels and diquat injection (*P* < 0.05). These results suggest that increasing dietary tryptophan levels could attenuate the oxidative stress of the liver in weaned piglets intraperitoneally injected with diquat via enhancing the antioxidant capacity.

## Background

Reactive oxygen species (ROS) include hydroxyl radical, superoxide anion and hydrogen peroxide, which are the unstable species with unpaired electron that can initiate oxidation [1]. Under the normal conditions, there is a dynamic equilibrium between the antioxidant capacity of the body and the production of ROS [2]. When ROS in the cells cannot be neutralized by the antioxidant defense mechanism, oxidative stress, induced the tissue damage and the oxidation of proteins, lipids and nucleic acids, will occur [3, 4]. For piglets, many factors, such as environmental factors, feeding oxidized diets, weaning and infection, can lead to oxidative stress that will produce the considerable economic loss for livestock producers.

Tryptophan is considered as the second-limiting amino acid in most corn-based diets of swine. Some previous studies have shown that dietary tryptophan levels could regulate feed intake, behavior, growth, immunity, protein synthesis and intestinal integrity of pigs [5–8]. Furthermore, increasing dietary tryptophan level could improve feed intake and growth performance of weanling pigs orally challenged with *Escherichia coli* K88 [9]. Shen et al. (2012) also reported that dietary tryptophan supplementation could decrease stress hormone secretion in pigs under social-mixing stress [10]. Dietary tryptophan supplementation also might alleviate inflammatory response, and thus increase the reproductive performance of the *Pseudorabies* virus-challenged female mice [11]. However, oxidative stress induced by diquat may increase the tryptophan metabolism of pigs in our previous study [12]. It is possible that the requirement of tryptophan is increased by oxidative stress.

Under heating, lipids of the feed may be oxidized to lipid peroxidation products that could induce the oxidative stress [13]. Diquat, a bipyridyl herbicide, could utilize molecular oxygen to generate superoxide anion radical, which is widely considered as an effective chemical agent for inducing oxidative stress [14]. Our previous study showed that, compared with feeding oxidized fish oil, injecting diquat could get the similar effect of oxidative stress, but it was more stable and effective [15]. Therefore, diquat injection was used to induce oxidative stress in this study.

In previous studies, dietary tryptophan supplementation can reduce the aggression and alleviate stress in pigs, rats and chickens [16–18], and some tryptophan metabolites and tryptophan-metabolizing enzymes, such as tryptophan 2,3-dioxygenase (TDO), have the antioxidant activity [19, 20]. Therefore, the current study was conducted to test the hypothesis that increasing dietary tryptophan levels could increase the antioxidant activity, and attenuate the oxidative stress induced by diquat in the weaned piglets.

## Materials and methods

### Animals and diets

The animal protocol for this study was approved by the Animal Care and Use Committee of Sichuan Agricultural University. A total of 36 PIC (Pig Improvement Company) barrows, weaned at 21 d of age, were housed individually in the stainless-steel metabolism cages (1.5 m × 0.7 m × 1.0 m). The diets were supplied 4 times daily at 08:00, 12:00, 16:00 and 20:00 h. The piglets had free access to drinking water. The room temperature was maintained at 25-28°C, and lighting was natural. At 08:00 h of d 1, 8 and 15, the body weight and feed intake of all piglets were measured, which were used to investigate average daily weight gain (ADG), average daily feed intake (ADFI) and feed efficiency.

Three isonitrogenous and isoenergetic diets were formulated to approximately meet National Research Council-recommended nutrient requirements for pigs weighing 10–20 kg (NRC 1998) [21], except for tryptophan (Table 1). However, National Research Council-recommended tryptophan requirement for pigs weighing 10–20 kg is 0.21% (NRC 1998) [21]. The three experimental diets were supplemented with varying amounts of L-tryptophan (98.0% purity; Evonik Degussa (China) Co., LTD., Beijing, China) to provide tryptophan levels of 0.18%, 0.30% and 0.45%. Alanine was added to the 0.18% and 0.30% tryptophan diets to obtain the same levels of total nitrogen as the 0.45% tryptophan diet.

**Table 1:** **Composition and nutrient levels of experimental diets**

	Trp 0.18%	Trp 0.30%	Trp 0.45%
Ingredients composition, %			
Corn	67.84	67.84	67.84
Extruded full-fat soybean	6.50	6.50	6.50
Soybean meal	4.50	4.50	4.50
Whey powder	6.00	6.00	6.00
Fish meal	5.50	5.50	5.50
Corn gluten meal	6.20	6.20	6.20
Corn starch	0.19	0.20	0.22
L-Lysine**·**HCL (78%)	0.47	0.47	0.47
L-Threonine (98.5%)	0.08	0.08	0.08
L-Alanine (98.5%)	0.31	0.17	0.00
L-Tryptophan (99%)	0.00	0.13	0.28
Dicalcium phosphate	1.00	1.00	1.00
Calcium carbonate	0.90	0.90	0.90
Sodium Chloride	0.30	0.30	0.30
Choline chloride	0.10	0.10	0.10
Vitamin premix^1^	0.03	0.03	0.03
Mineral premix^2^	0.08	0.08	0.08
Nutrient composition, %			
Digestible energy^3^, MJ/kg	14.22	14.22	14.22
Crude protein^4^	18.49	18.58	18.53
Total lysine^4^	1.22	1.20	1.19
Total methionine and cystine^4^	0.69	0.70	0.72
Total tryptophan^4^	0.19	0.29	0.42
Total threonine^4^	0.70	0.72	0.71
Calcium^3^	0.88	0.88	0.88
Phosphorus available^3^	0.49	0.49	0.49

Trp 0.18%, dietary tryptophan level 0.18%; Trp 0.30%, dietary tryptophan level 0.30%; Trp 0.45%, dietary tryptophan level 0.45%.

^1^Provided the following per kg of diet: Vitamin A, 2,800 IU; Vitamin D_3_, 320 IU; Vitamin E, 20 IU; Vitamin K_3_, 1 mg; Vitamin B_1_, 1.5 mg; Vitamin B_2_, 5 mg; Vitamin B_6_, 2 mg; Vitamin B_12_, 0.03 mg; Nicotonic, 20 mg; Pantothenic, 15 mg; Folic acid, 0.5 mg; Biotin, 0.1 mg.

^2^Provided the following per kg of diet: Fe (as FeSO_4_^.^7H_2_O), 100 mg; Cu (as CuSO_4_^.^5H_2_O), 6 mg; Zn (as ZnSO_4_^.^7H_2_O), 100 mg; Mn (MnSO_4_^.^H_2_O), 4 mg; Se (as Na_2_SeO_3_.5H_2_O), 0.3 mg; I (as KI), 0.14 mg.

^3^Calculated nutrient levels.

^4^Measured nutrient levels.

### Experimental design and sample collection

After 3 d of acclimatization, all piglets were weighed (6.82 ± 0.54 kg) and allotted randomly, on the basis of the initial body weights and origin of litters, to 1 of 6 treatments (n = 6/treatment) in a 3 × 2 factorial design experiment. Piglets were fed with the three diets containing the tryptophan levels of 0.18, 0.30 and 0.45% (namely two treatments per dietary group) in the first week. On d 8, all piglets were injected intraperitoneally with diquat (dibromide monohydrate, Chem Service, West Chester, PA) at a dose of 10 mg/kg body weight or the same amount of sterile 0.9% NaCl solution. Diquat was dissolved in a sterile 0.9% NaCl solution to a concentration of 10 mg/mL.

On d 15, after all pigs were food-deprived for 12 h, the blood samples of 20 mL were collected by anterior vena cava using vacutainer tubes coated with Na heparin (Axygen Biotechnology CO., LTD. Taizhou, China). Plasma was separated from the whole blood by centrifugation at 3,000 g for 10 min, and stored at −20°C until analysis. Following the blood sampling on d 15, all piglets were killed by an intracardial injection of Na pentobarbital (50 mg/kg body weight) and jugular exsanguinations. Then, the tissues of liver were immediately isolated, frozen in liquid nitrogen, and stored at −80°C until analysis.

### Chemical analyses

The crude protein in samples of diets was analyzed according to the Association of Official Analytical Chemists (AOAC 1995) [22] method (AOAC Method 988.05). The concentrations of amino acids in diets were determined following hydrolyzing in 6 N HCl at 110°C for 24 h, whereas content of sulfur-containing amino acids was measured after performic acid oxidation, and tryptophan content was determined after alkaline hydrolysis (AOAC method 994.12) [22]. Amino-acid analyses were performed by using an L-8800 Amino Acid Analyzer (Hitachi, Tokyo, Japan). The concentrations of plasma free amino acids were also determined using an L-8800 Amino Acid Analyzer (Hitachi, Tokyo, Japan), as previously described by Mao et al. (2012) [23]. Plasma urea was measured by an assay kit from Nanjing Jiancheng Biochemistry (Nanjing, China) according to the manufacturer’s instructions.

### The activities of antioxidant enzymes and the malondialdehyde (MDA) concentration analyses

The activities of superoxide dismutase (SOD) and glutathione peroxidase (GPx), and the concentration of MDA were determined using assay kits from Nanjing Jiancheng Biochemistry (Nanjing, China) according to the manufacturer’s instructions.

### Tryptophan 2,3-dioxygenase (TDO) activity in liver analysis

The TDO activity was determined according to a method previously described by Dairam et al. (2006) [24]. Briefly, the livers were homogenized with a solution containing 140 mmol/L KCl and 2.5 mmol/L NaOH by a glass-teflon hand held homogenizer. The homogenate was sonicated for a period of 2 min at 30 s intervals for completely releasing TDO from the cells. The 0.2 mol/L sodium phosphate buffer (pH 7.0) was used to make up the volume required to yield a 10% (v/v) homogenate. The whole procedure was conducted on ice as possible. An aliquot of 15 mL homogenate was added to a flask containing 12.5 mL water. The 100 μL of 2 μmol/L haematin was added to samples, and the sample was stirred for 1 min. The 2.5 mL of 0.03 mol/L L-tryptophan was added to the flask and gently stirred. The assay was conducted in triplicate. The 3 mL assay mixture was transferred to test tubes, stoppered under carbogen, and incubated for 1 h at 37°C in an oscillating water bath. The reaction was terminated with the addition of 2 mL of 0.9 mol/L trichloroacetic acid (TCA) to the reaction mixture, and incubated for 2–4 min. The reaction mixture was filtered through the filter paper. The 1.5 mL of 0.6 mol/L NaOH was added to 2.5 mL filtrate and vortexed. The kynurenine in the solution was measured at 365 nm spectrophotometrically using the molar extinction coefficient of kynurenine: ϵ = 4,540 L/mol · cm. The blank consisted of 2 mL of 0.9 mol/L TCA and 1.5 mL of 0.6 mol/L NaOH. The TDO activity was expressed as nmol/mg protein · h. The protein of livers was assayed by the Lowry method using bovine serum albumin as the standard [25].

### Statistical analysis

All data, expressed as means with their pooled standard errors, were analysed as a 3 × 2 factorial using the general linear model procedures of the Statistical Analysis System (Version 8.1; SAS Institute, Gary, NC). The factors in the models included the main effects of dietary tryptophan levels (0.18, 0.30 or 0.45%) and diquat injection (diquat or sterile 0.9% NaCl solution) as well as their interaction. Furthermore, all data were also analyzed using one-way ANOVA, followed by Ducan’s Multiple Range test of the Statistical Analysis System (Version 8.1; SAS Institute, Gary, NC). Statistical significance was considered at *P* < 0.05, tendency at *P* < 0.10.

## Results

### Effect of dietary tryptophan levels and diquat injection on the growth performance of the weaned piglets

During the first 7 days of trial, in the dietary 0.30% and 0.45% tryptophan level treatments compared with the dietary 0.18% tryptophan level treatment, ADG was 26% and 22% greater, respectively (*P* = 0.09, Table 2), and ADFI was 6% and 8% greater, respectively (*P* = 0.08, Table 2), but feed efficiency was 18% and 15% lower, respectively (*P* < 0.05, Table 2). However, intraperitoneal injection with diquat reduced the ADG (*P* < 0.05) and ADFI (*P* < 0.05) of piglets, and impaired the feed efficiency (*P* < 0.05) of piglets (Table 2). There were no interactions between dietary tryptophan levels and diquat injection with regard to the performance of the weaned piglets (Table 2).

**Table 2:** **Effects of dietary tryptophan levels and diquat injection on the growth performance of the weaned piglets**

Item	- diquat			+ diquat			SEM	***P***		
	Trp 0.18%	Trp 0.30%	Trp 0.45%	Trp 0.18%	Trp 0.30%	Trp 0.45%		Trp	diquat	Trp × diquat
1-7 d (n = 12)										
ADG, g	227	287	278				28	0.09		
ADFI, g	378	400	409				21	0.08		
Feed efficiency, g feed/g gain	1.74^a^	1.43^b^	1.48^b^				0.12	< 0.05		
8-14 d (n = 6)										
ADG, g	288^ab^	346^a^	336^a^	141^c^	224^abc^	170^bc^	42	0.27	< 0.05	0.88
ADFI, g	460^ab^	518^a^	512^a^	317^c^	427^abc^	368^bc^	43	0.17	< 0.05	0.79
Feed efficiency, g feed/g gain	1.72^b^	1.58^b^	1.58^b^	2.50^a^	2.04^ab^	2.22^ab^	0.21	0.35	< 0.05	0.76

+diquat, intraperitoneal injection diquat; − diquat, intraperitoneal injection of sterile 0.9% NaCl solution; Trp 0.18%, dietary tryptophan level 0.18%; Trp 0.30%, dietary tryptophan level 0.30%; Trp 0.45%, dietary tryptophan level 0.45%; ADG, average daily weight gain; ADFI, average daily feed intake.

^a, b, c^Mean values with unlike superscript letters were significantly different (*P* < 0.05).

(Mean values with their pooled standard errors).

### Effect of dietary tryptophan levels and diquat injection on the concentrations of tryptophan, large neutral amino acids (LNAA) and urea nitrogen in the plasma of the weaned piglets

Increasing dietary tryptophan levels enhanced the tryptophan concentration (*P* < 0.05) and tryptophan/LNAA (*P* < 0.05), and decreased the LNAA (*P* < 0.05) and urea nitrogen (*P* < 0.05) concentrations in the plasma of the weaned piglets (Table 3). However, intraperitoneal injection of diquat reduced the tryptophan concentration (*P* < 0.05) and tryptophan/LNAA (*P* < 0.05), and increased the LNAA (*P* = 0.05) and urea nitrogen (*P* < 0.05) concentrations in the plasma of the weaned piglets (Table 3). In addition, increasing dietary tryptophan levels attenuated the effects of diquat injection on the tryptophan (*P* = 0.08), LNAA (*P* < 0.05) and urea nitrogen (*P* = 0.06) concentrations in the plasma of the weaned piglets (Table 3). To the piglets that were injected diquat, in the plasma of the dietary 0.30% and 0.45% tryptophan level treatments compared with the dietary 0.18% tryptophan level treatment, the tryptophan concentrations were 38% and 52% greater, respectively (*P* < 0.05, Table 3), and the tryptophan/LNAA were 80% and 120% greater, respectively (*P* < 0.05, Table 3), but the LNAA concentrations were 23% and 27% lower, respectively (*P* < 0.05, Table 3), and the urea nitrogen were 36% and 33% lower, respectively (*P* < 0.05, Table 3).

**Table 3:** **Effects of dietary tryptophan levels and diquat injection on the concentrations of tryptophan, large neutral amino acids (LNAA) and urea nitrogen in the plasma of the weaned piglets**

Item	- diquat			+ diquat			SEM	***P***		
	Trp 0.18%	Trp 0.30%	Trp 0.45%	Trp 0.18%	Trp 0.30%	Trp 0.45%		Trp	diquat	Trp × diquat
Tryptophan, nmol/mL	45^b^	50^b^	61^a^	29^c^	40^b^	44^b^	4	< 0.05	< 0.05	0.08
LNAA, nmol/mL	497^b^	411^c^	453^bc^	582^a^	447^bc^	421^c^	17	< 0.05	0.05	< 0.05
Tryptophan/LNAA	0.09^b^	0.13^a^	0.14^a^	0.05^c^	0.09^b^	0.11^ab^	0.01	< 0.05	< 0.05	0.67
Plasma urea nitrogen, mg/L	95.12^c^	60.02^d^	64.27^d^	212.50^a^	135.02^b^	142.17^b^	7.90	< 0.05	< 0.05	0.06

+diquat, intraperitoneal injection diquat; − diquat, intraperitoneal injection of sterile 0.9% NaCl solution; Trp 0.18%, dietary tryptophan level 0.18%; Trp 0.30%, dietary tryptophan level 0.30%; Trp 0.45%, dietary tryptophan level 0.45%; LNAA, large neutral amino acids (sum of valine, methionine, isoleucine, leucine, phenylalanine, tyrosine and histidine).

^a, b, c^Mean values with unlike superscript letters were significantly different (*P* < 0.05).

(Mean values with their pooled standard errors, n = 6).

### Effects of dietary tryptophan levels and diquat injection on the activities of antioxidant enzymes and MDA in the plasma and liver of the weaned piglets

Increasing dietary tryptophan levels had no significant effect on the MDA concentration and the activities of SOD and GPx in the plasma and liver of the weaned piglets (Table 4). However, intraperitoneal injection of diquat decreased the SOD (*P* < 0.05) and GPx (*P* = 0.08) activities of the plasma and liver, and increased the MDA concentration of the plasma (*P* < 0.05) and liver (*P* = 0.09) in the weaned piglets (Table 4). In addition, there were no interactions between dietary tryptophan levels and diquat injection with regard to the MDA concentration and the activities of SOD and GPx in the plasma of the weaned piglets (Table 4), but increasing dietary tryptophan levels attenuated the effects of diquat injection on the MDA concentration (*P* = 0.06) and the activities of SOD (*P* = 0.09) and GPx (*P* = 0.05) in the liver of the weaned piglets (Table 4). To the piglets that were injected diquat, in the liver of the dietary 0.30% and 0.45% tryptophan level treatments compared with the dietary 0.18% tryptophan level treatment, the SOD activities were 10% and 12% greater, respectively, and the GPx activities were 18% and 31% greater, respectively, but the MDA concentrations were 17% and 9% lower (Table 4).

**Table 4:** **Effects of dietary tryptophan levels and diquat injection on the activities of antioxidant enzymes and the malondialdehyde concentration in the plasma and liver of the weaned piglets**

Item	- diquat			+ diquat			SEM	***P***		
	Trp 0.18%	Trp 0.30%	Trp 0.45%	Trp 0.18%	Trp 0.30%	Trp 0.45%		Trp	diquat	Trp × diquat
Plasma										
SOD, U/mL	142.51^a^	138.17^a^	135.20^a^	106.65^c^	118.94^bc^	115.55^bc^	6.76	0.81	< 0.05	0.47
GPx, U/mL	698.32^b^	811.76^a^	810.08^a^	665.55^b^	768.24^ab^	748.07^ab^	38.60	0.62	0.08	0.66
MDA, nmol/mL	2.43^b^	2.53^b^	2.48^b^	3.53^a^	3.58^a^	3.68^a^	0.21	0.89	< 0.05	0.94
Liver										
SOD, U/mg protein	564.43^a^	579.77^a^	595.80^a^	469.14^b^	516.04^ab^	527.69^ab^	31.64	0.74	< 0.05	0.09
GPx, U/mg protein	95.63^a^	95.71^a^	95.56^a^	76.04^b^	90.03^a^	99.44^a^	5.20	0.18	< 0.05	0.05
MDA, nmol/mg protein	1.60^a^	1.27^b^	1.56^a^	1.74^a^	1.44^ab^	1.59^a^	0.14	0.23	0.09	0.06

+diquat, intraperitoneal injection diquat; − diquat, intraperitoneal injection of sterile 0.9% NaCl solution; Trp 0.18%, dietary tryptophan level 0.18%; Trp 0.30%, dietary tryptophan level 0.30%; Trp 0.45%, dietary tryptophan level 0.45%; SOD, superoxide dismutase; GPx, glutathione peroxidase; MDA, malondialdehyde.

^a, b, c^Mean values with unlike superscript letters were significantly different (*P* < 0.05).

(Mean values with their pooled standard errors, n = 6).

### Effects of dietary tryptophan levels and diquat injection on the TDO activity in the liver of the weaned piglets

Increasing dietary tryptophan levels enhanced the TDO activity in the liver of the weaned piglets (*P* < 0.05, Table 5). The TDO activity in the liver was also increased by intraperitoneal injection of diquat (*P* = 0.07, Table 5). However, there is a synergistic role for regulating the TDO activity in the liver between dietary tryptophan levels and diquat injection (*P* < 0.05, Table 5). To the piglets that were injected diquat, in the liver of the dietary 0.30% and 0.45% tryptophan level treatments compared with the dietary 0.18% tryptophan level treatment, the TDO activities were 21% and 13% greater, respectively (*P* < 0.05, Table 5).

**Table 5:** **Effects of dietary tryptophan levels and diquat injection on tryptophan 2,3-dioxygenase activity in the liver of the weaned piglets**

Item	- diquat			+ diquat			SEM	***P***		
	Trp 0.18%	Trp 0.30%	Trp 0.45%	Trp 0.18%	Trp 0.30%	Trp 0.45%		Trp	diquat	Trp × diquat
TDO activity, nmol/mg protein·h	2.85^c^	3.24^b^	3.34^b^	3.07^bc^	3.72^a^	3.46^ab^	0.17	< 0.05	0.07	< 0.05

+diquat, intraperitoneal injection diquat; − diquat, intraperitoneal injection of sterile 0.9% NaCl solution; Trp 0.18%, dietary tryptophan level 0.18%; Trp 0.30%, dietary tryptophan level 0.30%; Trp 0.45%, dietary tryptophan level 0.45%; TDO, tryptophan 2,3-dioxygenase.

^a, b, c^Mean values with unlike superscript letters were significantly different (*P* < 0.05).

(Mean values with their pooled standard errors, n = 6).

## Discussion

Diquat is widely considered as an effective chemical agent for inducing oxidative stress [14], which of the major target organ is the liver [26]. Therefore, in order to determine whether increasing dietary tryptophan levels could attenuate oxidative stress in livers of piglets, a model by injecting diquat was used. In our previous study, intraperitoneal injection of diquat at a dose of 10 mg/kg body weight significantly decreased the plasma SOD and GPx activities, and significantly increased the plasma MDA concentration, but did not significantly affect the plasma catalase activity in the weaned piglets fed with the 0.18% tryptophan diet [12]. The present study also showed that intraperitoneal injection of diquat decreased the SOD and GPx activities of the plasma and liver, and increased the MDA concentration of the plasma and liver in the weaned piglets (Table 4), which is consistent with our previous studies [15, 27, 28]. These results indicated that the oxidative stress model induce by diquat injection was successful.

In this study, diquat injection increased the plasma LNAA and urea nitrogen concentrations, and decreased the plasma tryptophan concentration and growth performance in the weaned piglet (Tables 2 and 3), which is similar with the results of our previous study [12]. The decrease of the plasma tryptophan concentration could be due that diquat injection increased the TDO activity. However, the blood urea concentration may indirectly indicate the availability of amino acids for tissue growth [29]. Thus, intraperitoneal diquat injection could lead to the oxidative stress that impaired the protein metabolism in whole body, which increased the plasma LNAA concentration and decreased the availability of amino acids for tissue growth. Furthermore, our previous study did also show that, compared with control group and pair-fed group, diquat injection decreased the growth performance of pigs, and the growth performance of control group was higher than that of pair-fed group [12]. These studies showed that the decreasing growth performance of pigs induced by diquat injection could derive from the reduced nutrient supply and protein metabolic disturbance.

It has previously been shown that dietary adequate tryptophan level could increase the growth performance of piglets while the deficiency or excess of dietary tryptophan reduces those of piglets [5, 7, 8, 16, 30], and dietary tryptophan supplementation could also increase the growth performance of piglets though the experimental period was ≤10 d [7, 8, 10], which is consistent with the present results during the first 7 days of trial (Table 2). However, the previous studies did show that tryptophan supplementation in the tryptophan-deficient diet or under the stress condition could increase the plasma tryptophan concentration, decrease the plasma urea nitrogen and LNAA concentrations [16], and enhance the protein synthesis of liver, muscle and whole-body in pigs and rats [5, 31]. Therefore, during the first 7 days of trial, tryptophan supplementation increasing the growth performance could derive from the regulation of the protein metabolism. Additionally, in the present study, increasing dietary tryptophan levels also alleviated the effects of diquat injection on plasma tryptophan, LNAA and urea nitrogen concentrations (Table 3), which could determine that the availability of amino acids for tissue growth was improved by increasing dietary tryptophan levels in piglets that were intraperitoneally injected with diquat. However, during 7 d following diquat injection, increasing dietary tryptophan levels did not attenuated the effects of diquat injection on the growth performance of piglets in this study (Table 2), which could mainly be due that dietary tryptophan supplementation did not improve the reduced nutrient supply of pigs that were injected diquat.

SOD and GPx are the main antioxidant enzymes in mammals, which may reduce the accumulation of organic hydroperoxides and hydrogen peroxide in biological system [32–34]. The activities of SOD and GPx are commonly used to monitor the antioxidant capability of the body [35–37]. MDA, an end product of peroxidation of polyunsaturated fatty acids and related esters, is used as a marker of lipid peroxidation [38]. In the present study, increasing dietary tryptophan levels could attenuate the effects of diquat injection on the MDA concentration and the activities of SOD and GPx in the liver of the weaned piglets (Table 4), which could determine that increasing dietary tryptophan levels effectively alleviated the oxidative stress in the liver of the piglets that were intraperitoneally injected with diquat.

TDO, is a sister enzyme of indoleamine 2,3-dioxygenase that is a rate-limiting enzyme of the tryptophan metabolism in the liver of rats, may catalyze the oxygenation of tryptophan into kynurenines via using a superoxide anion as an oxidative co-factor [20, 24]. Thus, it can be considered as a genuine antioxidant enzyme. Furthermore, some tryptophan metabolites, including 5-hydroxytryptophan and tryptophan metabolites of the kynurenine pathway (such as 3-hydroxyanthranilic acid and 3-hydroxykynurenine), have the antioxidant capacity [19]. Our previous study has shown that diquat injection could increase the TDO mRNA level in the liver of piglets [12]. In the present study, the TDO activity in the liver was also enhanced by intraperitoneal injection of diquat (Table 5). This could be part of the self-adjustment ability in the liver under oxidative stress that induced by diquat injection. However, increasing dietary tryptophan levels could further promote the TDO activity of the liver that was induced by diquat injection in weaned piglets (Table 5), which could result in the increasing typtophan metabolism through kynurenine pathway, and enhance the 3-hydroxyanthranilic acid and 3-hydroxykynurenine. These also suggested that increasing dietary tryptophan levels efficiently alleviated the oxidative stress in the liver of piglets intraperitoneally injected with diquat partially via regulating the TDO activity and some non-enzymatic antioxidant in the liver.

In this study, the three experimental diets contained tryptophan levels of 0.18%, 0.30% and 0.45% by supplementing L-tryptophan. During the experiment, via determining all indexes, the diets containing 0.30% and 0.45% tryptophan levels could be better than the one containing 0.18% tryptophan level. However, although there were no significant difference in all indexes between the diets containing 0.30% and 0.45% tryptophan levels, the growth performance, antioxidant capacity, plasma urea nitrogen and TDO activity of pigs that were fed the diets containing 0.30% tryptophan level were better than those of pigs that were fed the diets containing 0.45% tryptophan level.

## Conclusions

This study further indicates that intraperitoneal injection of diquat decreased the growth performance of the weaned piglets via oxidative stress that could result in the reduced nutrient supply and nutrient metabolism disturbance. Under oxidative stress induced by diquat, dietary tryptophan supplementation efficiently alleviated the oxidative stress in the liver of the weaned piglets via increasing antioxidant capacity. Additionally, based on the results of this study, dietary tryptophan level of the 10–20 kg piglet should be increased to 0.30% if radical oxidative stress occurs.

## Abbreviations

ADFI: Average daily feed intake
ADG: Average daily weight gain
GPx: Glutathione peroxidase
LNAA: Large neutral amino acids
MDA: Malondialdehyde
ROS: Reactive oxygen species
SOD: Superoxide dismutase
TCA: Trichloroacetic acid
TDO: Tryptophan 2,3-dioxygenase
