# Correction to: The effect of dietary tryptophan levels on oxidative stress of liver induced by diquat in weaned piglets

**DOI:** 10.1186/s40104-021-00631-w

**Published:** 2021-10-21

**Authors:** Xiangbing Mao, Mei Lv, Bing Yu, Jun He, Ping Zheng, Jie Yu, Quyuan Wang, Daiwen Chen

**Affiliations:** 1 Animal Nutrition Institute, Sichuan Agricultural University, Xinkang Road 46#, Ya’an, Sichuan Province 625014 People’s Republic of China; 2 Key Laboratory of Animal Disease-Resistance Nutrition, Ministry of Education, Ya’an, 625014 People’s Republic of China

**Correction to: J Anim Sci Biotechnol. 5, 49 (2014)**

**https://doi.org/10.1186/2049-1891-5-49**

Following publication of the original article [1], it was found that the Table 3 (the data of Plasma urea nitrogen) and Table 4 (the data of Plasma SOD and MDA) are incorrect.

**Table 3:** Effects of dietary tryptophan levels and diquat injection on the concentrations of tryptophan, large neutral amino acids (LNAA) and urea nitrogen in the plasma of the weaned piglets

Item	- diquat			+ diquat			SEM	*P*		
	Trp 0.18%	Trp 0.30%	Trp 0.45%	Trp 0.18%	Trp 0.30%	Trp 0.45%		Trp	diquat	Trp × diquat
Tryptophan, nmol/mL	45^b^	50^b^	61^a^	29^c^	40^b^	44^b^	4	< 0.05	< 0.05	0.08
LNAA, nmol/mL	497^b^	411^c^	453^bc^	582^a^	447^bc^	421^c^	17	< 0.05	0.05	< 0.05
Tryptophan/LNAA	0.09^b^	0.13^a^	0.14^a^	0.05^c^	0.09^b^	0.11^ab^	0.01	< 0.05	< 0.05	0.67
Plasma urea nitrogen, mg/L	108.46^b^	75.30^c^	76.21^c^	134.53^a^	67.11^c^	74.75^c^	5.90	< 0.05	0.29	< 0.05

+ diquat, intraperitoneal injection diquat; − diquat, intraperitoneal injection of sterile 0.9% NaCl solution; Trp 0.18%, dietary tryptophan level 0.18%; Trp 0.30%, dietary tryptophan level 0.30%; Trp 0.45%, dietary tryptophan level 0.45%; *LNAA*, large neutral amino acids (sum of valine, methionine, isoleucine, leucine, phenylalanine, tyrosine and histidine). ^a, b, c^Mean values with unlike superscript letters were significantly different (*P* <  0.05). (Mean values with their pooled standard errors, *n* = 6)

**Table 4:** Effects of dietary tryptophan levels and diquat injection on the activities of antioxidant enzymes and the malondialdehyde concentration in the plasma and liver of the weaned piglets

Item	- diquat			+ diquat			SEM	*P*		
	Trp 0.18%	Trp 0.30%	Trp 0.45%	Trp 0.18%	Trp 0.30%	Trp 0.45%		Trp	diquat	Trp × diquat
Plasma										
SOD, U/mL	131.55	130.60	131.37	121.26	135.07	129.65	7.13	0.71	0.69	0.64
GPx, U/mL	698.32^b^	811.76^a^	810.08^a^	665.55^b^	768.24^ab^	748.07^ab^	38.60	0.62	0.08	0.66
MDA, nmol/mL	2.28	2.48	2.43	2.41	2.28	2.31	0.26	0.89	0.60	0.97
Liver										
SOD, U/mL	564.43^a^	579.77^a^	595.80^a^	469.14^b^	516.04^ab^	527.69^ab^	31.64	0.74	< 0.05	0.09
GPx, U/mL	95.63^a^	95.71^a^	95.56^a^	76.04^b^	90.03^a^	99.44^a^	5.20	0.18	< 0.05	0.05
MDA, nmol/mL	1.60^a^	1.27^b^	1.56^a^	1.74^a^	1.44^ab^	1.59^a^	0.14	0.23	0.09	0.06

+ diquat, intraperitoneal injection diquat; − diquat, intraperitoneal injection of sterile 0.9% NaCl solution; Trp 0.18%, dietary tryptophan level 0.18%; Trp 0.30%, dietary tryptophan level 0.30%; Trp 0.45%, dietary tryptophan level 0.45%; *SOD*, superoxide dismutase; *GPx,* Glutathione peroxidase, *MDA,* Malondialdehyde. ^a, b, c^Mean values with unlike superscript letters were significantly different (*P* < 0.05). (Mean values with their pooled standard errors, *n* = 5 or 6)

The correct tables are provided below:

The original paper has been updated.

## References

[1] Mao X (2014). The effect of dietary tryptophan levels on oxidative stress of liver induced by diquat in weaned piglets. J An Sci Biotechnol.

